# Transmembrane TNF-alpha promotes chemoresistance in breast cancer cells

**DOI:** 10.1038/s41388-018-0221-4

**Published:** 2018-03-21

**Authors:** Zunyue Zhang, Guohong Lin, Yujing Yan, Xiang Li, Yibing Hu, Jing Wang, Bingjiao Yin, Yaqun Wu, Zhuoya Li, Xiang-Ping Yang

**Affiliations:** 1 Department of Immunology, School of Basic Medicine, Tongji Medical College, Huazhong University of Science and Technology, Wuhan, 430030 China; 2 Department of Thyroid and Breast Surgery, Tongji Hospital, Tongji Medical College, Huazhong University of Science and Technology, Wuhan, 430030 China

## Abstract

Chemoresistance remains a major obstacle to successful treatment of breast cancer. Although soluble tumor necrosis factor-α (sTNF-α) has been implicated in mediating drug-resistance in human cancers, whether transmembrane tumor necrosis factor-α (tmTNF-α) plays a role in chemoresistance remains unclear. Here we found that over 50% of studied patients expressed tmTNF-α at high levels in breast cancer tissues and tmTNF-α expression positively correlated with resistance to anthracycline chemotherapy. Alteration of tmTNF-α expression changed the sensitivity of primary human breast cancer cells and breast cancer cell lines to doxorubicin (DOX). Overexpression of N-terminal fragment (NTF) of tmTNF-α, a mutant form with intact intracellular domain of tmTNF-α to transmit reverse signals, induced DOX-resistance. Mechanistically, the tmTNF-α/NTF-ERK-GST-π axis and tmTNF-α/NTF-NF-κB-mediated anti-apoptotic functions were required for tmTNF-α-induced DOX-resistance. In a xenograft mouse model, the combination of tmTNF-α suppression with chemotherapy significantly enhanced the efficacy of DOX. Our data indicate that tmTNF-α mediates DOX-resistance through reverse signaling and targeting tmTNF-α may be beneficial for the treatment of DOX-resistant breast cancer.

## Introduction

Despite many advances in the treatment of breast cancer, the prognosis for patients with metastatic disease remains poor. Cytotoxic chemotherapy is of limited benefit and chemoresistance remains a major obstacle. The anthracycline antibiotics containing doxorubicin (DOX) [1–3] are used in multiple solid tumors, including bladder cancer, small cell lung cancer, and breast cancer and in hematological malignancies, such as acute myeloid and lymphoblastic lymphoma [4–6]. Multiple mechanisms have been proposed in the resistance of DOX-induced cytotoxicity, including increased drug efflux through upregulating expression of transporters such as the P-glycoprotein (P-gp) and multidrug resistance protein-1 (MRP-1) [7, 8], altered enzymatic activity of glutathione transferase, mutations in DNA of topoisomerase (TOP) and increased expression of anti-apoptotic proteins such as Bcl-2 [9, 10].

Tumor necrosis factor-α (TNF-α) has two bioactive forms: transmembrane TNF-α (tmTNF-α) and secretory TNF-α (sTNF-α). tmTNF-α is cleaved by a metalloproteinase TNF-α-converting enzyme (TACE) to generate sTNF-α, leaving an N-terminal fragment (NTF) of TNF-α on the cell surface [11]. TNF-α can be produced by tumor cells, infiltrating immune cells and stroma cells in tumor microenvironment. Patients with multiple advanced cancers have elevated TNF-α expression in biopsies and in the plasma [12]. sTNF-α is well known to be involved in all steps of tumor development, including tumorigenesis, proliferation, angiogenesis, metastasis, and subverting the immune responses [12, 13]. Furthermore, sTNF-α induces resistance to BRAF inhibitors in melanoma cells [14] and to cisplatin chemotherapy in malignant pleural mesothelioma [15]. So far, most studies focus on the role of sTNF-α in tumorigenesis and tumor development, less is known about the functions of tmTNF-α in cancer, especially in chemoresistance.

tmTNF-α on the membrane functions not only as a receptor but also as a ligand depending on the context [16, 17]. As a ligand, tmTNF-α expressed on effector cells, such as macrophages and natural killer (NK) cells, executes anti-tumor activity [18, 19]. Conversely, as a receptor expressed on tumor cells, tmTNF-α facilitates growth of leukemia stem cells via its outside-to-inside signal transduction (reverse signaling) [20]. Overexpression of NTF of tmTNF-α transmits only reverse signaling but not forward signaling [21]. We previously showed that tmTNF-α is expressed at high levels in primary breast cancers, but not in normal breast tissue [22]. tmTNF-α-positive MDA-MB-231 breast cancer cells are resistant to sTNF-α-induced cytotoxicity, but tmTNF-α-negative MCF-7 breast cancer cells are sensitive. Ectopic expression of the NTF of tmTNF-α in MCF-7 leads to constitutive activation of NF-κB and resistance to sTNF-α-mediated cytotoxicity [21], suggesting a possible role of tmTNF-α-mediated reverse signaling in constitutive activation of NF-κB in tumor cells. NF-κB activation is associated with multiple processes in tumor biology, including chemoresistance [23, 24]. We speculated that tmTNF-α expressed by breast cancer cells might promote tumor development and chemoresistence via reverse signaling.

In this study, we found that the expression of tmTNF-α correlated with the disease severity and DOX-resistance. The drug-resistance was associated with tmTNF-α-mediated reverse signaling. Suppressing tmTNF-α expression increased the sensitivity of breast cancer cells towards DOX both in vitro andin vivo. Our data indicate that tmTNF-α serves not only as a tumor biomarker but also as an attractive target for the treatment of DOX-resistant breast cancer.

## Results

### tmTNF-α is expressed in human primary breast cancer cells and associated with adverse clinical features

To investigate the contribution of tmTNF-α in the pathogenesis of breast cancer, we used an in house anti-tmTNF-α monoclonal antibody without cross-reaction to sTNF-α to detect tmTNF-α expression in primary tumoral and peritumoral tissues of invasive breast ductal carcinoma patients (*n* = 105) by immunohistochemistry (IHC) [21]. We found that the expression of tmTNF-α was undetectable in peritumor breast tissue, whereas 63.8% of breast cancers contained tumor cells that expressed tmTNF-α at high levels (++/+++) (Supplementary Figures 1a and b). Notably, tmTNF-α expression positively correlated with tumor size (>2 cm), incidence of metastasis and HER2 expression (Table 1). In contrast, tmTNF-α expression inversely correlated with sex hormone receptors (ER and PR) (Table 1). Of note, tmTNF-α expression was 84% in 25 cases of triple-negative breast cancer (TNBC) (Table 1), implying tmTNF-α as a possible marker and target for treatment of TNBC. Together, the clinical data suggest that tmTNF-α is closely linked to the development of breast cancer including promotion of tumor growth and metastasis.

**Table 1:** A relationship of tmTNF-α expression and clinical characteristics of ductal breast cancer (*n* = 105)

Characteristics	Patients	tmTNF-α expression					*p*-value
		Negative	Low	Middle	High	Positive rate (%)	
Age (years)							
≤50	63	16	8	26	13	74.60%	
>50	42	10	4	20	8	76.19%	
Grade							
I	9	3	2	3	1	66.67%	
II	88	22	9	37	20	75.00%	
III	8	1	1	6	0	87.50%	
Tumor Size (cm)							
≤2	35	15	6	12	2	57.14%	0.002
>2	70	11	6	35	18	83.58%	
Node status							
Negative (0)	50	17	7	18	8	66.00%	0.0028
Positive (≥1)	55	9	5	29	12	83.64%	
Estrogen Receptor							
Negative	45	5	5	21	14	88.89%	0.0108
Positive	60	21	7	25	7	65.00%	
Progesterone Receptor							
Negative	52	7	5	26	14	86.54%	0.0068
Positive	53	19	7	20	7	64.15%	
HER 2							
Negative	67	24	8	24	11	64.18%	0.0003
Positive	38	2	4	22	10	94.74%	
Triple-negative	25	4	2	12	7	84.00%	
Non-TNBC	80	22	10	34	14	72.50%	
Total	105	26	12	46	21		

Non-TNBC: Non-triple-negative breast cancer

### Knockdown of tmTNF-α expression suppresses proliferation, clonogenicity and metastasis of breast cancer cells

**Fig. 1:**
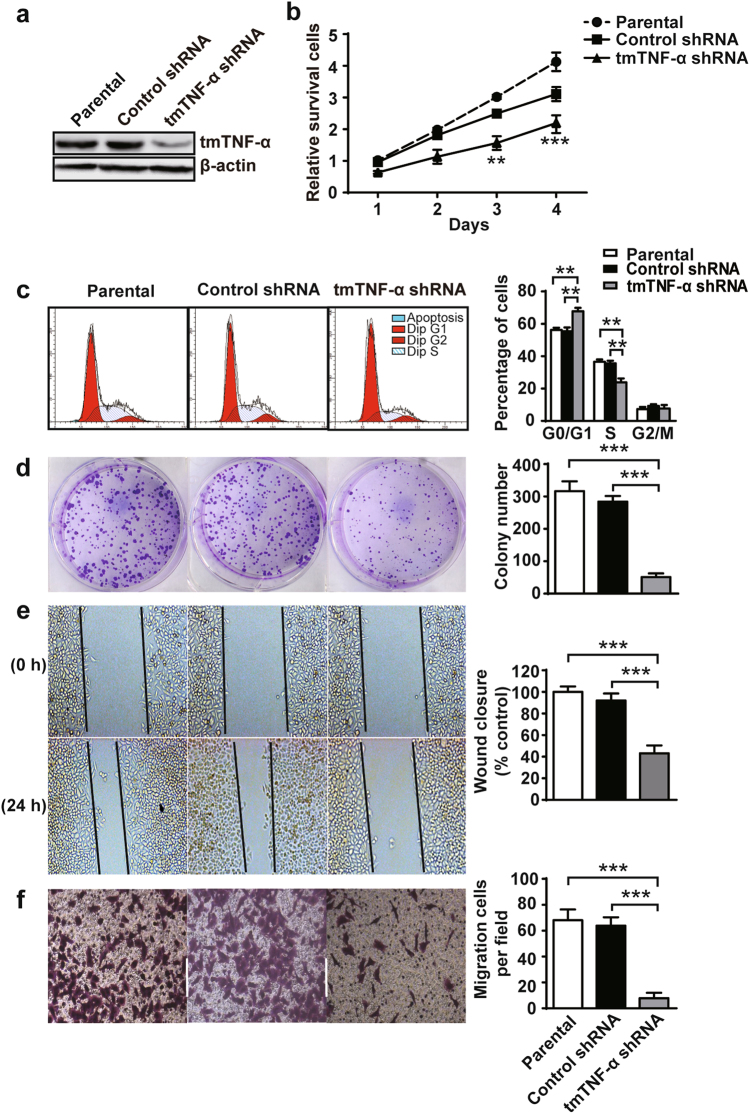
tmTNF-α promotes proliferation, clonogenicity, migration and invasion of MDA-MB-231. MDA-MB-231 breast cancer cells were stably transfected with control or tmTNF-α shRNA. **a** tmTNF-α expression determined by Western blot in parental or shRNA-transfected MDA-MB-231 cells. **b** Cell proliferation was detected after incubation for indicated time points by a CCK8 assay. **c** Quantification of DNA content by flow cytometry for analysis of cell cycle following propodium iodide staining. **d** Clonogenicity of parental or shRNA-transfected MDA-MB-231 cells was detected by colony formation assay after a 24-h culture. **e** Cell migration was evaluated using a wound-healing assay after 0 or 24-h culture. **f** Number of invasive cells cross through matrigel and membrane porous over 24 h. **c**–**f** Representative images on the left, and the quantitative data on the right are presented as mean ± SEM of three independent experiments. **p < 0.01, ***p < 0.001

To determine whether tmTNF-α affects the proliferative and metastatic capacities of breast cancer cells, MDA-MB-231 human breast cancer cell line with high expression of tmTNF-α was stably transfected with shRNA to knockdown the membrane molecule (Fig. 1a). shRNA knockdown of tmTNF-α expression significantly reduced cell numbers (Fig. 1b) and led to a significant decrease in the number of tumor cells in S phase, with a concomitant increase in the proportion of cells in G0/G1 (Fig. 1c). In addition, a significant reduction in the number of colonies in tmTNF-α knockdown cells was observed (Fig. 1d). Using an in vitro wound-healing assay and matrigel assay, we found that downregulation of tmTNF-α in MDA-MB-231 cells resulted in a prominent decrease in migrated and invaded cells, compared with parental and control shRNA groups (Fig. 1e, f), suggesting that tmTNF-α facilitates migration and invasion of the tumor cells. Taken together, tmTNF-α, consistent with our clinical correlates, enhanced the proliferation, clonogenicity and metastasis of breast cancer cells.

### tmTNF-α mediates DOX-resistance in human primary breast cancer cells and breast cancer cell lines via reverse signaling

**Fig. 2:**
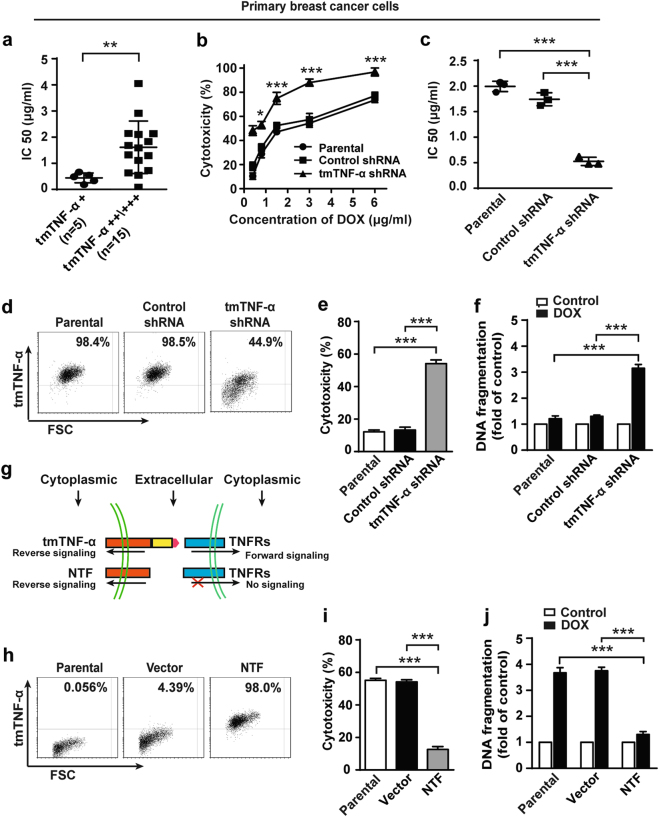
tmTNF-α is required for DOX-resistance of breast cancer. **a** Primary human breast cancer cells were isolated from patients with low (*n* = 5) or high (*n* = 15) expression of tmTNF-α and incubated with DOX for 72 h. IC50 values of DOX-mediated cytotoxicity was determined by ATP-TCA. **b**, **c** Primary breast cancer cells from three patients were transfected with control or tmTNF-α shRNA for 48 h. DOX was added in indicated concentrations and incubated for 72 h. **b** Dose dependent DOX-induced cytotoxicity determined by ATP-TCA. **c** IC50 values of DOX to primary cancer cells or those transfected with shRNA. **d** FACS analysis of tmTNF-α expression in parental or shRNA stably transfected MDA-MB-231 cells. **e**, **f** Cytotoxicity and DNA fragmentation of parental or shRNA-transfected MDA-MB-231 cells treated with 3 μM DOX for 24 h detected by MTT assay and ELISA, respectively. **g** Scheme of NTF-tmTNF-α-mediated reverse signaling without delivering forward signaling. **h** FACS analysis of NTF expression in parental, or empty vector or NTF-transfected MCF-7 cells. **i** and **j** Cytotoxicity and DNA fragmentation of parental or NTF-transfected MCF-7 cells treated with 3 μM DOX for 24 h. Data are represented as mean ± SEM of three independent experiments. *p < 0.05, **p < 0.01, ***p < 0.001

DOX is one of the most widely used cytotoxic chemotherapeutic drugs in the treatment of breast cancer. We previously showed that tmTNF-α expressing tumor cells are resistant to sTNF-α-induced apoptosis [21, 25]. We wondered if tmTNF-α also mediates DOX-resistance. To this end, we compared the sensitivity to DOX of primary breast cancer cells among 20 patients with different levels of tmTNF-α using ATP-tumor chemo-sensitivity assay. Primary cancer cells were divided into low (+) or high (++/+++) expression of tmTNF-α and incubated for 72 h with DOX. The 5 cases with low expression of tmTNF-α had significantly lower IC50 scores (median 0.44 μg/ml) compared with the 15 cases that had high expression of tmTNF-α (median 1.61 μg/ml) (Fig. 2a). These indicate that high expression of tmTNF-α was associated with the drug-resistance. To confirm this, we knocked down tmTNF-α expression in primary breast cancer cells from 3 patients highly expressing the molecule (Supplementary Figures 2a and b). As expected, suppression of tmTNF-α expression significantly increased DOX-induced cytotoxicity (Fig. 2b), resulting in a marked decrease of IC50 from 1.99 ± 0.10 μg/ml to 0.53 ± 0.08 μg/ml (Fig. 2c).

To further substantiate the contribution of tmTNF-α in DOX-resistance, we compared the sensitivity of tmTNF-α-positive MDA-MB-231 cells and tmTNF-α-negative MCF-7 to DOX toxicity (Supplementary Figure 2c). We found that MCF-7 cells were more sensitive to DOX-induced cell death (Supplementary Figure 2d) and DNA fragmentation (Supplementary Figure 2e) compared with MDA-MB-231 cells. Knockdown of tmTNF-α expression by shRNA (Fig. 2d) rendered MDA-MB-231 cells sensitive to the drug, showing significantly enhanced DOX-induced cytotoxicity (Fig. 2e) and DNA fragmentation (Fig. 2f).

As tmTNF-α functions not only as a ligand to deliver forward signaling by binding to receptors but also as a receptor to transmit reverse signaling into the tmTNF-α bearing cells [25], we stably transfected the NTF of tmTNF-α in TNF-α-negative MCF-7 cells to exclude the effect of sTNF-α and forward signaling of tmTNF-α (Fig. 2g, h). Overexpressing the NTF of tmTNF-α in MCF-7 cells resulted in a conversion from sensitivity to resistance to DOX-induced cytotoxicity (Fig. 2i) and DNA fragmentation (Fig. 2j). Altogether, these data demonstrate that expression of tmTNF-α confers the DOX-resistance in both primary breast cancer cells and breast cancer cell lines.

### tmTNF-α upregulates GST-π expression through ERK activation to promote DOX-resistance

A variety of molecules including glutathione S-transferase-π (GST-π), P-gp, O6-methylguanine–DNA methyltransferase (MGMT), MRP-1, breast cancer resistance protein (BCRP) and TOP2a have been shown to mediate DOX-resistance [26–28]. To explore the molecular mechanism underlying tmTNF-α-mediated drug-resistance, we determined whether knockdown of tmTNF-α expression in MDA-MB-231 cells or forced expression of the NTF of tmTNF-α in MCF-7 cells altered the expression of these molecules measured by flow cytometry. We found no significant effect of altering tmTNF-α on the expression of MGMT, P-gp, TOP2a or MRP-1, although expression of the first three were higher in MCF-7 compared with MDA-MB-231 cells (Supplementary Figures 3a-3d). By contrast, GST-π and BCRP were expressed at higher levels in MDA-MB-231 cells than those in MCF-7 cells. tmTNF-α knockdown was associated with a significant reduction in GST-π and BCRP expression in MDA-MB-231 and overexpression of the tmTNF-α NTF upregulated the expression of the both molecules in MCF-7 cells (Supplementary Figures 3e and 3f), suggesting that GST-π and BCRP are downstream targets of tmTNF-α.

**Fig. 3:**
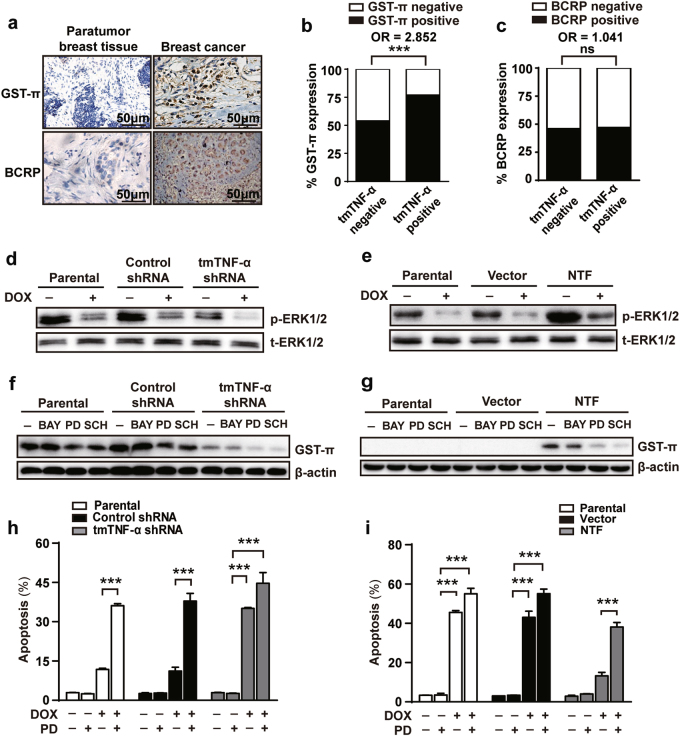
ERK-GST-π is involved in tmTNF-α-mediated DOX-resistance. **a** Representative IHC images of GST-π and BCRP expression in paratumor and breast cancer tumor sections. **b**, **c** Histogram showing co-expression rate of GST-π or BCRP with tmTNF-α in tumor tissues of 105 patients. **d**, **e** Immunoblotting analysis of phosphorylation levels of ERK1/2 in parental or shRNA-transfected MDA-MB-231 cells (**d**) and in parental or NTF-transfected MCF-7 cells (**e**) after treatment with or without 3 μM DOX for 24 h. **f**, **g** Immunoblotting analysis of GST-π expression in parental or shRNAs-transfected MDA-MB-231 cells and in parental or NTF-transfected MCF-7 cells treated with 10 μM BAY117082 (BAY), 10 μM PD98059 (PD) or 1 μM SCH772984 (SCH) for 24 h. **h**, **i** A 24-h DOX (3 μM)-induced apoptosis in parental or shRNA-transfected MDA-MB-231 cells and in parental or NTF-transfected MCF-7 cells pretreated with 10 μM PD98059 for 30 min using Annexin V/PI staining. Data are represented as mean ± SEM of three independent experiments. ***p < 0.001

Next, we compared the expression of GST-π and BCRP in tumoral and peritumoral tissues (*n* = 105) using IHC and analyzed their association with tmTNF-α expression (Fig. 3a). Although 77.22% of patients with tmTNF-α expression were GST-π positive compared with only 52% in the tmTNF-α negative group (Fig. 3b), showing a positive correlation of expression between these two molecules (OR 2.852, *p* = 0.001), tmTNF-α expression had no association with BCRP (Fig. 3c). It has been reported that ERK pathway can be activated by reverse signaling of tmTNF-α [29]. Consistent with this, there was enhanced ERK phosphorylation in MDA-MB-231 compared with MCF-7 cells. Furthermore, knockdown of tmTNF-α expression by shRNA blocked ERK phosphorylation in MDA-MB-231 (Fig. 3d), conversely, ectopic expression of NTF promoted ERK phosphorylation in MCF-7 cells (Fig. 3e). In all cases ERK phosphorylation could be inhibited by treatment with DOX.

To investigate if tmTNF-α-induced GST-π expression was dependent on ERK signaling, we incubated transfected cell lines with the ERK inhibitors PD98059 and SCH772984. Both inhibitors could partially suppress GST-π transcription and protein expression in tmTNF-α expressing MDA-MB-231 cells (Fig. 3f and Supplementary Figure 3g) or in MCF-7 cells that were transfected with NTF (Fig. 3g and Supplementary Figure 3h). However, the NF-κB inhibitor BAY117082 had no effect on tmTNF-α or NTF-induced GST-π transcription and protein expression in MDA-MB-231 cells or in MCF-7 cells, respectively. The ability of ERK inhibitors to suppress GST-π expression was associated with an increase in DOX-induced apoptosis and cytotoxicity in tmTNF-α expressing MDA-MB-231 cells (Fig. 3h and Supplementary Figures 3i and 3k) or in NTF-expressing MCF-7 cells (Fig. 3i and Supplementary Figures 3j and 3l).

### GST-π contributes to tmTNF-α-mediated DOX-resistance through detoxification and ERK activation

**Fig. 4:**
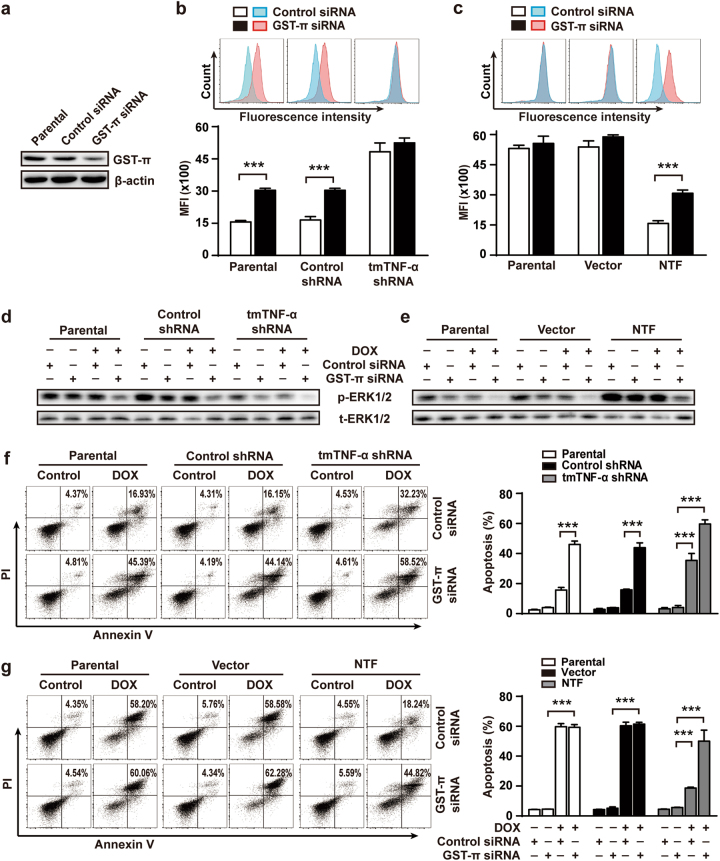
Knockdown of GST-π expression blocked tmTNF-α-mediated DOX-resistance. Parental or shRNAs-transfected MDA-MB-231 cells and parental or NTF-transfected MCF-7 cells were transfected with 50 μM control or GST-π siRNA for 48 h and then treated with DOX (3 μM) for another 24 h. **a** Immunoblotting analysis of GST-π expression in MDA-MB-231 cells. **b**, **c** DOX fluorescence intensity was determined by FACS. **d**, **e** Immunoblotting analysis of phosphorylation levels of ERK1/2. **f**, **g** The apoptosis was determined by PI/Annexin V staining. Representative cytogram on the right and quantitative data on the left. Data are represented as mean ± SEM of three independent experiments. ***p < 0.001

GST-π can detoxify a variety of electrophilic compounds including exogenous xenobiotics such as mutagens and anticancer agents [30]. Next we asked if tmTNF-α promoted GST-π-mediated DOX detoxification. Knockdown of tmTNF-α in MDA-MB-231 cells resulted in enhanced DOX fluorescence intensity, whereas overexpression of NTF in tmTNF-α-negative MCF-7 cells inhibited intracellular DOX MFI, suggesting that tmTNF-α can detoxify DOX via reverse signaling. However, suppression of GST-π expression by siRNA (Fig. 4a) partially blocked the effect of tmTNF-α or NTF, increasing the intracellular amounts of DOX (Fig. 4b, c), but had no effect in tmTNF-α knockdown MDA-MB-231 cells or in tmTNF-α-negative MCF-7 cells. These data indicate that tmTNF-α induced DOX-resistance partially through GST-π-mediated detoxification.

As GST-π can also regulate MAPK pathways in addition to detoxification, affecting chemoresistance [31, 32], we asked if GST-π affects tmTNF-α-induced ERK activation. We found that tmTNF-α or NTF-induced obvious phosphorylation of ERK1/2 (Fig. 4d, e) and resistance to DOX-induced apoptosis (Fig. 4f, g), which were significantly blocked by silence of GST-π expression. Notably, GST-π knockdown also suppressed ERK1/2 phosphorylation in the tmTNF-α-silenced or negative cells, indicating an effect of GST-π on tmTNF-α-independent ERK activation. However, GST-π knockdown had no effect on DOX-induced apoptosis in tmTNF-α-negative MCF-7 cells (Fig. 4g), implying that tmTNF-α-induced GST-π expression was responsible for tmTNF-α-mediated DOX-resistance. Altogether, the results suggest that GST-π contributes to tmTNF-α-induced DOX-resistance through detoxification and ERK activation.

### NF-κB is necessary for tmTNF-mediated DOX-resistance

**Fig. 5:**
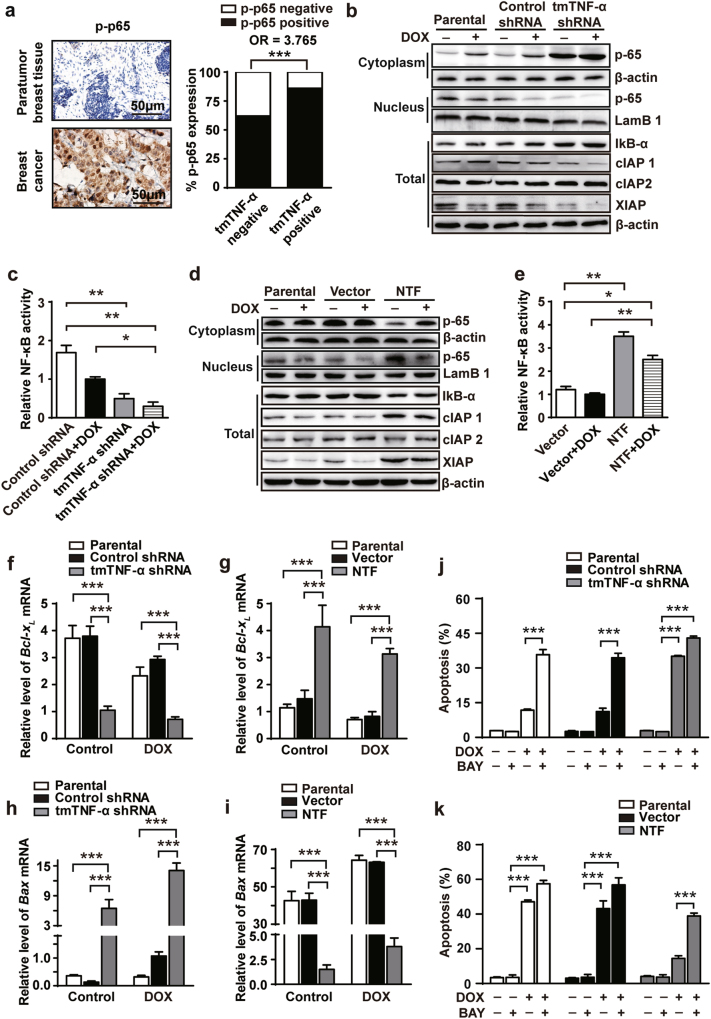
NF-κB is required for tmTNF**-**α-mediated DOX-resistance. **a** Representative IHC images of phosphorylation of NF-κB p65 in paratumor and breast cancer tumors, and histogram showing co-expression rate of phosphorylated NF-κB p65 and tmTNF-α in tumors of 105 patients. (**b**-**i**) Parental or shRNA-transfected MDA-MB-231 cells and parental or NTF-transfected MCF-7 cells were treated with or without 3 μM DOX for 24 h. (**b** and **d**) Representative immunoblotting of levels of IκB-α, cIAP1, cIAP2, and XIAP in total protein and translocation of NF-κB p65 from cytoplasmic fraction to nuclear fraction. LamB1 or β-actin served as a nuclear protein or a cytoplasmic/total protein loading control. **c**, **e** NF-κB activity was detected by ELISA. **f**–**i** Real-time PCR analysis of Bcl-X_L_ (**f** and **g**) and BAX (**h** and **i**). **j**, **k** A 24 h-DOX (3 μM)-induced apoptosis was determined in parental or shRNA-transfected MDA-MB-231 cells (**j**) and in parental or NTF-transfected MCF-7 cells (**k**) pretreated with 10 μM BAY117082 (BAY) for 30 min using Annexin V/PI staining. Data are represented as mean ± SEM of three independent experiments. *p < 0.05, **p < 0.01, ***p < 0.001

Our previous study showed that tmTNF-α induces constitutive activation of NF-κB in cancer cells through reverse signaling [21, 25]. We examined the phosphorylation of NF-κB p65 in primary cancer tissues by IHC. We found a significantly elevated proportion of tmTNF-α^+^ cells positive for phospho-p65 compared with tmTNF-α^−^ cells, showing a positive correlation (OR 3.765, *p* < 0.001) of expression between these two molecules (Fig. 5a). Consistent with this, we found elevated constitutive NF-κB activation in tmTNF-α-positive MDA-MB-231 cells (Fig. 5b, c), but not in tmTNF-α-negative MCF-7 cells (Fig. 5d, e). Knockdown of tmTNF-α in MDA-MB-231 cells resulted in enhanced IκB-α expression, reduced translocation of p65 from cytoplasm into the nucleus (Fig. 5b), decreased NF-κB activity (Fig. 5c) and downregulated expression of anti-apoptotic NF-κB targeted genes cIAP1 and XIAP (Fig. 5b). Conversely, overexpression of NTF in MCF-7 cells led to degradation of IκB-α, increased translocation of p65 in the nucleus (Fig. 5d), enhanced NF-κB activity (Fig. 5e), and upregulated expression of cIAP1 and XIAP (Fig. 5d). DOX has been reported to induce apoptosis by downregulation of Bcl-X_L_, an anti-apoptotic molecule, and upregulation of BAX, a pro-apoptotic molecule, in breast cancer cells [33–35]. Knockdown of tmTNF-α expression in MDA-MB-231 cells significantly reduced, but ectopic expression of NTF in MCF-7 cells markedly enhanced, transcription (Fig. 5f, g) and protein expression (Supplementary Figures 4a and b) of Bcl-X_L_ regardless of the presence of DOX. Conversely, transcription and protein expression of BAX was increased by knockdown of tmTNF-α in MDA-MB-231 cells (Fig. 5h and Supplementary Figure 4a) but decreased in NTF-expressing MCF-7 cells (Fig. 5i, Supplementary Figure 4b).

To test if NF-κB activation is required for tmTNF-α-mediated DOX-resistance, we suppressed NF-κB activation with BAY117082 and found that the inhibitor reversed the DOX-resistance of tmTNF-α-positive MDA-MB-231 cells (Fig. 5j and Supplementary figures 4c and e) and of NTF-overexpressing MCF-7 cells (Fig. 5k and Supplementary figures 4d and f). Altogether, these results demonstrate that NF-κB activation is required for tmTNF-α-mediated drug-resistance.

### Knockdown of tmTNF-α expression enhances the therapeutic efficacy of DOX in a xenograft mouse model

**Fig. 6:**
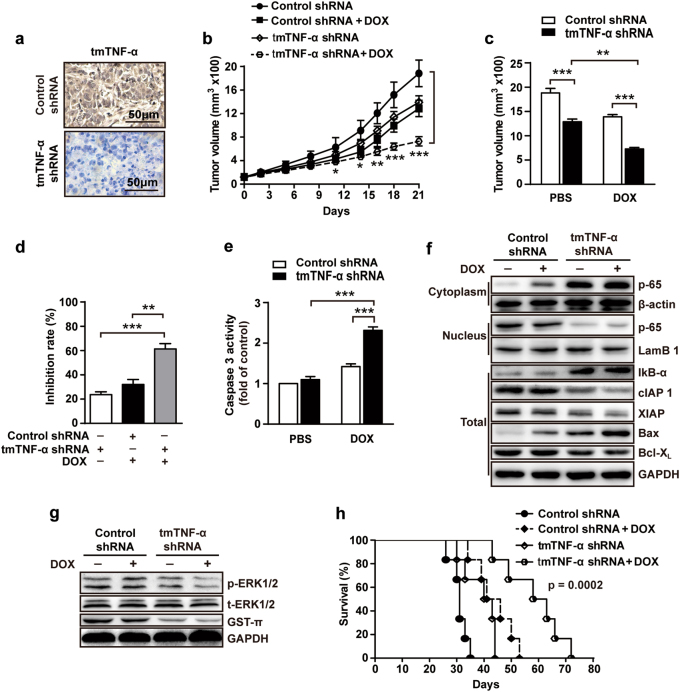
Knockdown of tmTNF-α expression enhances the therapeutic efficacy of DOX in vivo. 1 × 10^6^ MDA-MB-231 cells transfected with control or tmTNF-α shRNA were injected into mammary fat pad of nude mice. When tumors reached 100 mm^3^, the mice were treated intraperitoneally with DOX (4 mg/kg) or PBS, once a week for three weeks (*n* = 6 each group). **a** Representative images of tmTNF-α expression in tumor tissue section detected by IHC. **b** Growth curves of tumors after tumor cell inoculation. **c** Tumor sizes at the end point (day 21) of experiments. **d** Inhibition rate of tumor growth. **e** Caspase 3 activity in tumor tissues at day 21. **f** Immunoblotting of levels of IκB-а, cIAP1, cIAP2, XIAP, BAX, and Bcl-X_L_ in total protein and translocation of NF-κB p65 from cytoplasmic fraction to nuclear fraction of tumor tissues. **g** Immunoblotting of levels of phosphorylation of ERK and GST-π in tumor tissues. **h** Kaplan–Meier survival curves of mice after tumor cell inoculation (*n* = 6). Data are represented as mean ± SEM. *p < 0.05, **p < 0.01, ***p < 0.001

Next, we asked if targeting tmTNF-α affects tumorigenic ability of breast cancer cells and sensitivity to DOX treatment in vivo. To test this, we injected MDA-MB-231 cells stably transfected with control shRNA or tmTNF-α shRNA into mammary fat pad of the female athymic nude mice. After tumor formation (about 100 mm^3^), the animals were received intraperitoneal injection of either PBS or DOX (4 mg/kg) once a week for 3 weeks. We found that tmTNF-α was highly expressed in tumor tissues transfected with control shRNA, but markedly inhibited by transfection of tmTNF-α shRNA (Fig. 6a). Mice that received tmTNF-α-knockdown MDA-MB-231 cells had significantly smaller tumors compared with animals that received MDA-MB-231 cells transfected with control shRNA (Fig. 6b, c), while the combination of tmTNF-α inhibition and DOX treatment resulted in much more pronounced suppression of tumor growth (61% after 21 days, Fig. 6b–d) than treatment with either DOX (32%) or silence of tmTNF-α (24%). As DOX exerts effect on tumor cell by induction of apoptosis [36–39], we examined caspase 3 activity. Although knockdown of tmTNF-α expression alone did not activate caspase 3, it significantly increased DOX-induced activation of caspase 3 (Fig. 6e), indicating a synergetic effect of tmTNF-α inhibition on DOX-induced apoptosis.

We next examined whether inhibition of tmTNF-α could suppress ERK and NF-κB pathways in vivo. shRNA knockdown of tmTNF-α expression enhanced IκB-α levels, blocked p65 nucleus translocation, and reduced expression of cIAP1, XIAP, and Bcl-X_L_ in the tumor tissues (Fig. 6f). In contrast, the expression of proapoptotic protein BAX was increased upon tmTNF-α knockdown (Fig. 6f). In addition, knockdown of tmTNF-α expression suppressed ERK phosphorylation in tumor tissues and downregulated GST-π expression (Fig. 6g). Finally, we investigated the effect of all of these differences on the survival of the animals after inoculation followed by DOX treatment. Mice that received MDA-MB-231 cells transfected with control shRNA only had a significantly shorter median survival time (about 35 days). Either tmTNF-α knockdown or DOX treatment prolonged the median survival time of tumor-bearing mice to 44 days and 53 days, respectively. Of note, the combination of tmTNF-α knockdown and DOX treatment significantly extended the survival time of mice (72 days) (Fig. 6h). These results demonstrate again that tmTNF-α contributes to drug-resistance in breast cancer and knockdown of tmTNF-α expression renders tumor cells more sensitive to DOX treatment.

## Discussion

**Fig. 7:**
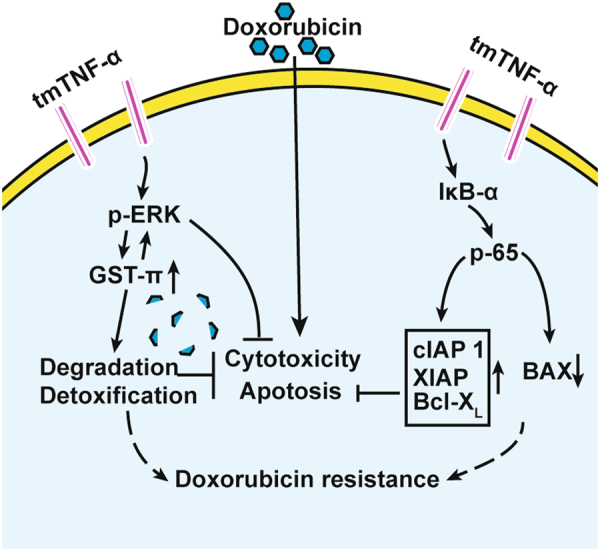
tmTNF-α induces DOX-resistance of breast cancer cells via its reverse signaling. tmTNF-α can be overexpressed in breast cancer cells and tends to induce chemoresistance. tmTNF-α constitutively activates both ERK and NF-κB pathways through reverse signaling. tmTNF-α promotes GST-π expression via ERK pathway, leading to degradation and detoxification of DOX in tumor cells, and tmTNF-α-induced GST-π in turn further activates ERK pathway, forming a positive feedback to promote DOX-resistance. tmTNF-α can also upregulate antiapoptotic molecules via NF-κB pathway and downregulate proapoptotic molecule, resulting in survival and anti-apoptosis of tumor cells. Targeting tmTNF-α may sensitize tumor cells to chemotherapy

Here, we found that high expression of tmTNF-α is linked to adverse clinical features of breast cancer and associated with tumor resistance to DOX. We uncovered a novel mechanism that tmTNF-α on breast cancer cells activates the ERK/GST-π axis and NF-κB pathway via reverse signaling to mediate the resistance to DOX (Fig. 7). Targeting tmTNF-α significantly improves the sensitivity of tumor cells to the chemotherapy.

Our previous study showed that tmTNF-α is expressed in breast cancer tissue, to a lesser extent in hyperplasia and atypical hyperplasia, but not in paratumor breast tissue [22]. In this study, we enlarged the size of patients with invasive breast ductal carcinoma and found that over 60% patients (*n* = 105) with ductal breast cancer expressed tmTNF-α at high levels, which was positively correlated with tumor size and lymph node metastasis. This is accordance with our previous study on acute leukemia showing that tmTNF-α correlated with poor risk stratification, extramedullary infiltration and adverse clinical parameters [20]. We found a high expression of tmTNF-α in 84% of 25 cases with TNBC, which lack surface biomarker and are highly lethal. Thus, tmTNF-α may be a potential target in the treatment of TNBC [40], although further analysis with an expanded sample size is warranted.

We found that tmTNF-α confers a gross pro-tumor activity, as knockdown of tmTNF-α expression in MDA-MB-231 significantly inhibited tumor cell proliferation, colony formation, migration and invasion. Our previous study showed that anti-tmTNF-α antibody depleted tmTNF-α highly expressing tumor cells by antibody-dependent cell-mediated cytotoxicity and the surviving tumor cells with low level of tmTNF-α became sensitive to sTNF-α-mediated cytotoxicity [22]. In this study, we found that tmTNF-α confers DOX chemoresistance in both primary breast cancer cells and breast cancer cell lines. Knockdown of tmTNF-α expression leads to a switch of tumor cells from resistance to sensitivity to DOX in vitro and in vivo. NTF-tmTNF-α lacks one part of extracellular domain (sTNF-α part) and cannot bind TNFR to transmit forward signals, while it has an intact intracellular domain to deliver reverse signals. The fact that ectopic expression of the tmTNF-α NTF in MCF-7 cells renders drug-sensitive cells resistant to DOX-induced cytotoxicity suggests that tmTNF-α-mediated reverse signaling might be a general mechanism employed by different types of cancer cells to induce resistance to DOX.

GST-π, a highly inducible phase II detoxification enzyme, is regulated by transcriptional factors including activating protein-1 (AP-1) and nuclear factor erythroid-2-related factor 2 (Nrf2), and functions in detoxifying multiple anti-cancer drugs including chlorambucil, cyclophosphamide, cisplatin, DOX and mitoxantrone [41, 42]. Our results demonstrated that tmTNF-α or NTF expressed by breast cancer cells promoted GST-π expression that reduced intracellular DOX amounts by detoxification and degradation, inducing DOX-resistance. However, silence of GST-π increased intracellular DOX amounts in tmTNF-α-positive or NTF-overexpressing cancer cells and partially reversed tmTNF-α-induced DOX-resistance, indicating a contribution of GST-π detoxification to tmTNF-α-induced DOX-resistance. As the ERK and NF-κB pathways can be activated by reverse signaling of tmTNF-α [25, 29], we used inhibitors for the both pathways and found that inhibition of ERK pathway, but not the NF-κB pathway, partially blocked tmTNF-α-induced GST-π expression and significantly reversed tmTNF-α or NTF-induced DOX-resistance. Consistently, Lin et al. [43] reported that GST-π can be upregulated by phosphorylation of ERK2 and activating Nrf2 in response to methionine restriction. In addition, our results showed that tmTNF-α-induced GST-π expression could in turn further activate ERK pathway, creating a positive feedback loop to promote DOX-resistance, as either silence of GST-π expression or inhibition of ERK pathway reversed tmTNF-α-induced DOX-resistance. This suggests that the tmTNF-α-ERK-GST-π axis may play a role in DOX chemoresistance.

Although sTNF-α/NF-κB pathway has been shown to mediate drug-resistance in different human cancers via TNFR [44–46], here we uncovered that tmTNF-α-mediated NF-κB activation via its reverse signaling is critical for DOX-resistance of breast cancer. First, we found that NF-κB was constitutively activated not only in over 80% tmTNF-α-positive primary cancers, but also in tmTNF-α-expressing breast cancer cell line. Second, knockdown of tmTNF-α expression resulted in suppression of NF-κB activation in MDA-MB-231 cells. Third, the most important, ectopic expression of tmTNF-α NTF constitutively activates NF-κB pathway without binding to TNFR, suggesting that tmTNF-α reverse signaling is an intrinsic activator for NF-κB. This is similar to our previous study showing that leukemia cell line expressing tmTNF-α constitutively activates NF-κB via its reverse signaling, inducing resistance to sTNF-α-mediated cytotoxicity [25]. Montagut et al. [47] reported that breast cancer patients with NF-κB activation only had a 20% clinical response rate, while patients with undetected NF-κB activation had a 91% response rate to neoadjuvant chemotherapy. tmTNF-α induced upregulation of NF-κB targeted antiapoptotic genes XIAP, cIAP1 and Bcl-X_L_, but downregulation of proapoptotic molecule BAX, which facilitates tumor cells evading apoptosis as another mechanism underlying tmTNF-α-induced chemoresistance. Therefore, consistent with a report showing inhibition of NF-κB pathway sensitizes human cancer cells to DOX [48], the NF-κB inhibitor BAY117082 blocked DOX-resistance of tmTNF-α-expressing MDA-MB-231 cells and restored the drug sensitivity of NTF-expressing MCF-7 cells.

A large body of evidence has been shown that combination of antibody-based targeted therapy and chemotherapy significantly improves outcome of tumor patients. For example, trastuzumab, a humanized monoclonal antibody binding to HER2, combined with chemotherapy approximately halves the recurrence risk, increasing 10-year overall survival [49]. We previously showed that targeting tmTNF-α with monoclonal antibody efficiently inhibited breast cancer tumor growth and induced apoptosis [22]. Therefore, combination of TNF-α antibody and chemotherapy may be a successful strategy for treatment of chemo-resistant advanced breast cancer.

## Materials and methods

More detailed information is described in Supplementary Materials and methods.

### Patients

Breast cancer tissues were collected from 105 patients with ductal breast cancer based on the pathological diagnosis in Tongji Hospital, Huazhong University of Science and Technology. Paratumoral breast tissues were taken 2 cm adjacent to breast tumors from 20 patients. The primary breast cancer cells isolated from fresh tumor tissues of 20 patients were used to detect their sensitivity to DOX. The study was approved by the Clinical Research Committee of Tongji Medical College, Huazhong University of Science and Technology, and informed consent was obtained from all subjects before conducting this study.

### Cell culture

Human breast carcinoma cell lines MCF-7 and MDA-MB-231 were purchased from American Type Culture Collection (ATCC, USA). Cells were cultured at 37 °C in 5% CO_2_ in RPMI-1640 medium (Life Technologies, USA) supplemented with 10% heat-inactivated, pyrogen-free fetal calf serum (FCS, Sijiqing, Hangzhou, China), 1 mM sodium pyruvate, 2 mM l-glutamine, 100 U/mL penicillin and 100 mg/ml streptomycin.

### Transfection of NTF, shRNA and siRNA

NTF of tmTNF-α stably transfected MCF-7 cell line was generated, as described previously [21]. To knockdown tmTNF-α expression, MDA-MB-231 cells were transfected with 1.5 μg plasmids encoding either the TNF-α-specific short hairpin RNA (shRNA) 5′-TTGGTGACCAACTGTCACTCATTGCTGAG-3′ or control shRNA (OriGene, USA) using Lipofectamine 2000 transfect reagent (Invitrogen, USA). Cells were selected with 2 µg/ml puromycin for 2 weeks and sub-cloned by dilution at 0.3 cells/well in 96-well microliter plates with 1 µg/ml puromycin.

To knockdown GST-π expression, the breast cancer cells were transfected with 50 μM control siRNA or siRNA against GST-π (CCTGGTGGACATGGTGAAT) (RiboBio, China) for 48 h using Lipofectamine 2000 transfect reagent (Invitrogen, USA).

### Cell drug sensitivity assay

Chemosensitivity of primary breast cancer cells was evaluated with the ATP-tumor chemosensitivity assay (ATP-TCA) kit (Beijing jinzijing Biotech, Beijing, China). Fresh tumors were obtained during surgery, minced into smaller fragments (1 mm^3^), resuspended in 5–10 ml sterile digestive enzyme reagent and incubated for 2–3 h at 37 °C in a 5% CO_2_ incubator. After centrifugation at 400 × *g* for 10 min, primary breast cancer cells were collected. For knockdown of tmTNF-α, cells were transfected with 1.5 μg tmTNF-α shRNA or control shRNA using lipofectamine 2000 transfect reagent (Invitrogen, USA) according to manufacturer’s protocol. After 48 h transfection, cells were seeded in a 96-well microplate. DOX was added in triplicate at five different doses of 12.5, 25, 50, 100, and 200% of a standard peak plasma concentration (PPC). The PPC value of DOX was 3 μg/ml. For each concentration, adding 100 μl of ATP inhibitor for maximum inhibition served as a positive control and complete assay medium only as a negative control. Plates were incubated for 72 h at 37 °C with 95% humidity in a 5% CO_2_ incubator. The cells were then lysed by the addition of 50 μl tumor cell extraction reagent, followed by adding 50 μl luciferin-luciferase reagent to each well. Luminescence was measured by a microplate luminometer (Berthold Diagnostic Systems, Hamburg, Germany).

For testing drug sensitivity of cell lines, Annexin V staining (BD Pharmingen, USA) and MTT assay were used. Cells were treated with 3 μM or different concentrations of DOX (Sigma, USA) for 24 h, in the absence and presence of PD98059 (10 μM), SCH772984 (1 μM) or BAY117082 (10 μM). The inhibitors were added 30 mins prior to the addition of DOX. For MTT assay, cells were lysed with 0.1 ml 100% DMSO (Sigma-Aldrich, USA) after stained with glucose-PBS containing 0.45 mg/ml MTT (Sigma-Aldrich) for 4 h. The OD value at 570 nm was measured on a microplate reader (Tecan, Grodig, Austria). Cytotoxicity was calculated by the following formula: Cytotoxicity (%) = (1−OD_sample_/OD_control_)×100%. For apoptosis, FITC Annexin V Apoptosis Detection Kit (BD Pharmingen, USA) was used to test the apoptosis rate. After washing with cold PBS, cells were resuspended in binding buffer and stained with Annexin V and PI for 15 min at room temperature. Apoptosis was analyzed by flow cytometry (Becton Dickinson, San Jose, CA) using BD FACS Diva software.

### Xenotransplantation of MDA-MB-231 cells into nude mice

Six-week-old female BALB/c nude mice were purchased from Beijing HFK Bioscience Company (Beijing, China). Mice were bred in a specific pathogen-free barrier facility, and animal experiments were approved by the Animal Care and Ethics Committee of Tongji Medical College, Huazhong University of Science and Technology. Mice were randomly grouped and subcutaneously injected with 1 × 10^6^ MDA-MB-231 cells stably transfected with tmTNF-α shRNA or control shRNA in 100 μl PBS into the right mammary fat pads. Tumor size was measured every 3 days with microcalipers in blind manner and calculated using the following equation: length×width^2^×π/6. When tumors reached 100 mm^3^, the mice were randomly grouped and treated intraperitoneally with DOX (4 mg/kg) or PBS, once a week for three weeks (*n* = 6 each group).

The survival curves were estimated using the Kaplan–Meier method, and the differences in survival among the 4 groups were compared using the log-rank test.

### Statistical analysis

Data are represented as the mean ± SEM (of three independent experiments in vitro). The differences were analyzed using one-way or two-way ANOVA test with GraphPad software. The clinical data were evaluated by the *χ*^2^ test and odds ratio (95% confidence intervals) with SPSS software. A *p*-value <0.05 was considered statistically significant.

## Electronic supplementary material

Supplementary Data(DOC 2116 kb)

## References

[1] Arcamone F, Cassinelli G, Fantini G, Grein A, Orezzi P, Pol C (2000). Adriamycin, 14-hydroxydaunomycin, a new antitumor antibiotic from S. peucetius var. caesius. Reprinted from Biotechnology and Bioengineering, Vol. XI, Issue 6, Pages 1101–1110 (1969). Biotechnol Bioeng.

[2] Mackey JR, Martin M, Pienkowski T, Rolski J, Guastalla JP, Sami A (2013). Adjuvant docetaxel, doxorubicin, and cyclophosphamide in node-positive breast cancer: 10-year follow-up of the phase 3 randomised BCIRG 001 trial. Lancet Oncol.

[3] Martin M, Ruiz A, Ruiz Borrego M, Barnadas A, Gonzalez S, Calvo L (2013). Fluorouracil, doxorubicin, and cyclophosphamide (FAC) versus FAC followed by weekly paclitaxel as adjuvant therapy for high-risk, node-negative breast cancer: results from the GEICAM/2003-02 study. J Clin Oncol: Off J Am Soc Clin Oncol.

[4] Dalpiaz O, al Rabi N, Galfano A, Martignoni G, Ficarra V, Artibani W (2003). Small cell carcinoma of the bladder: a case report and a literature review. Arch Esp Urol.

[5] Filipits M, Pohl G, Stranzl T, Kaufmann H, Ackermann J, Gisslinger H (2003). Low p27Kip1 expression is an independent adverse prognostic factor in patients with multiple myeloma. Clin Cancer Res: Off J Am Assoc Cancer Res.

[6] Verma S, Younus J, Stys-Norman D, Haynes AE, Blackstein M, Members of the Sarcoma Disease Site Group of Cancer Care Ontario’s Program in Evidence-Based C. (2008). Meta-analysis of ifosfamide-based combination chemotherapy in advanced soft tissue sarcoma. Cancer Treat Rev.

[7] Higgins CF (2007). Multiple molecular mechanisms for multidrug resistance transporters. Nature.

[8] Meng H, Liong M, Xia T, Li Z, Ji Z, Zink JI (2010). Engineered design of mesoporous silica nanoparticles to deliver doxorubicin and P-glycoprotein siRNA to overcome drug resistance in a cancer cell line. ACS Nano.

[9] Johnson NA, Slack GW, Savage KJ, Connors JM, Ben-Neriah S, Rogic S (2012). Concurrent expression of MYC and BCL2 in diffuse large B-cell lymphoma treated with rituximab plus cyclophosphamide, doxorubicin, vincristine, and prednisone. J Clin Oncol: Off J Am Soc Clin Oncol.

[10] Pendleton M, Lindsey RH, Felix CA, Grimwade D, Osheroff N (2014). Topoisomerase II and leukemia. Ann N Y Acad Sci.

[11] Kriegler M, Perez C, DeFay K, Albert I, Lu SD (1988). A novel form of TNF/cachectin is a cell surface cytotoxic transmembrane protein: ramifications for the complex physiology of TNF. Cell.

[12] Balkwill F (2009). Tumour necrosis factor and cancer. Nat Rev Cancer.

[13] Balkwill F (2006). TNF-alpha in promotion and progression of cancer. Cancer Metastas- Rev.

[14] Gray-Schopfer VC, Karasarides M, Hayward R, Marais R (2007). Tumor necrosis factor-alpha blocks apoptosis in melanoma cells when BRAF signaling is inhibited. Cancer Res.

[15] Gordon GJ, Mani M, Mukhopadhyay L, Dong L, Yeap BY, Sugarbaker DJ (2007). Inhibitor of apoptosis proteins are regulated by tumour necrosis factor-alpha in malignant pleural mesothelioma. J Pathol.

[16] Black RA, Rauch CT, Kozlosky CJ, Peschon JJ, Slack JL, Wolfson MF (1997). A metalloproteinase disintegrin that releases tumour-necrosis factor-alpha from cells. Nature.

[17] Daniel D, Wilson NS (2008). Tumor necrosis factor: renaissance as a cancer therapeutic?. Curr Cancer Drug Targets.

[18] Kresse M, Latta M, Kunstle G, Riehle HM, van Rooijen N, Hentze H (2005). Kupffer cell-expressed membrane-bound TNF mediates melphalan hepatotoxicity via activation of both TNF receptors. J Immunol.

[19] Yu M, Shi W, Zhang J, Niu L, Chen Q, Yan D (2009). Influence of reverse signaling via membrane TNF-alpha on cytotoxicity of NK92 cells. Eur J Cell Biol.

[20] Zhou X, Zhou S, Li B, Li Q, Gao L, Li D (2015). Transmembrane TNF-alpha preferentially expressed by leukemia stem cells and blasts is a potent target for antibody therapy. Blood.

[21] Yan D, Qin N, Zhang H, Liu T, Yu M, Jiang X (2009). Expression of TNF-alpha leader sequence renders MCF-7 tumor cells resistant to the cytotoxicity of soluble TNF-alpha. Breast Cancer Res Treat.

[22] Yu M, Zhou X, Niu L, Lin G, Huang J, Zhou W (2013). Targeting transmembrane TNF-alpha suppresses breast cancer growth. Cancer Res.

[23] Baldwin AS (2012). Regulation of cell death and autophagy by IKK and NF-kappaB: critical mechanisms in immune function and cancer. Immunol Rev.

[24] Li F, Zhang J, Arfuso F, Chinnathambi A, Zayed ME, Alharbi SA (2015). NF-kappaB in cancer therapy. Arch Toxicol.

[25] Zhang H, Yan D, Shi X, Liang H, Pang Y, Qin N (2008). Transmembrane TNF-alpha mediates “forward” and “reverse” signaling, inducing cell death or survival via the NF-kappaB pathway in Raji Burkitt lymphoma cells. J Leukoc Biol.

[26] Kalinina EV, Chernov NN, Saprin AN, Kotova YN, Remizov VI, Shcherbak NP (2007). Expression of genes for redox-dependent glutathione S-transferase isoforms GSTP1-1 and GSTA4-4 in tumor cell during the development doxorubicin resistance. Bull Exp Biol Med.

[27] Sharom FJ (2008). ABC multidrug transporters: structure, function and role in chemoresistance. Pharmacogenomics.

[28] Zajchowski DA, Karlan BY, Shawver LK (2012). Treatment-related protein biomarker expression differs between primary and recurrent ovarian carcinomas. Mol Cancer Ther.

[29] Kirchner S, Boldt S, Kolch W, Haffner S, Kazak S, Janosch P (2004). LPS resistance in monocytic cells caused by reverse signaling through transmembrane TNF (mTNF) is mediated by the MAPK/ERK pathway. J Leukoc Biol.

[30] Townsend DM, Tew KD (2003). The role of glutathione-S-transferase in anti-cancer drug resistance. Oncogene.

[31] Huang G, Mills L, Worth LL (2007). Expression of human glutathione S-transferase P1 mediates the chemosensitivity of osteosarcoma cells. Mol Cancer Ther.

[32] Brantley-Finley C, Lyle CS, Du L, Goodwin ME, Hall T, Szwedo D (2003). The JNK, ERK and p53 pathways play distinct roles in apoptosis mediated by the antitumor agents vinblastine, doxorubicin, and etoposide. Biochem Pharmacol.

[33] Gahl RF, He Y, Yu S, Tjandra N (2014). Conformational rearrangements in the pro-apoptotic protein, Bax, as it inserts into mitochondria: a cellular death switch. J Biol Chem.

[34] Garner TP, Reyna DE, Priyadarshi A, Chen HC, Li S, Wu Y (2016). An autoinhibited dimeric form of BAX regulates the BAX activation pathway. Mol Cell.

[35] Sharifi S, Barar J, Hejazi MS, Samadi N (2015). Doxorubicin changes Bax /Bcl-xL Ratio, caspase-8 and 9 in breast cancer cells. Adv Pharm Bull.

[36] Casares N, Pequignot MO, Tesniere A, Ghiringhelli F, Roux S, Chaput N (2005). Caspase-dependent immunogenicity of doxorubicin-induced tumor cell death. J Exp Med.

[37] Myers CE, McGuire WP, Liss RH, Ifrim I, Grotzinger K, Young RC (1977). Adriamycin: the role of lipid peroxidation in cardiac toxicity and tumor response. Science.

[38] Pang B, Qiao X, Janssen L, Velds A, Groothuis T, Kerkhoven R (2013). Drug-induced histone eviction from open chromatin contributes to the chemotherapeutic effects of doxorubicin. Nat Commun.

[39] Surova O, Zhivotovsky B (2013). Various modes of cell death induced by DNA damage. Oncogene.

[40] Foulkes WD, Smith IE, Reis-Filho JS (2010). Triple-negative breast cancer. N Engl J Med.

[41] Louie SM, Grossman EA, Crawford LA, Ding L, Camarda R, Huffman TR (2016). GSTP1 is a driver of triple-negative breast cancer cell metabolism and pathogenicity. Cell Chem Biol.

[42] Sweeney C, McClure GY, Fares MY, Stone A, Coles BF, Thompson PA (2000). Association between survival after treatment for breast cancer and glutathione S-transferase P1 Ile105Val polymorphism. Cancer Res.

[43] Lin AH, Chen HW, Liu CT, Tsai CW, Lii CK (2012). Activation of Nrf2 is required for up-regulation of the pi class of glutathione S-transferase in rat primary hepatocytes with L-methionine starvation. J Agric Food Chem.

[44] Acharyya S, Oskarsson T, Vanharanta S, Malladi S, Kim J, Morris PG (2012). A CXCL1 paracrine network links cancer chemoresistance and metastasis. Cell.

[45] Chen Q, Boire A, Jin X, Valiente M, Er EE, Lopez-Soto A (2016). Carcinoma-astrocyte gap junctions promote brain metastasis by cGAMP transfer. Nature.

[46] Wang LC, Okitsu CY, Zandi E (2005). Tumor necrosis factor alpha-dependent drug resistance to purine and pyrimidine analogues in human colon tumor cells mediated through IKK. J Biol Chem.

[47] Montagut C, Tusquets I, Ferrer B, Corominas JM, Bellosillo B, Campas C (2006). Activation of nuclear factor-kappa B is linked to resistance to neoadjuvant chemotherapy in breast cancer patients. Endocr Relat Cancer.

[48] Tapia MA, Gonzalez-Navarrete I, Dalmases A, Bosch M, Rodriguez-Fanjul V, Rolfe M (2007). Inhibition of the canonical IKK/NF kappa B pathway sensitizes human cancer cells to doxorubicin. Cell Cycle.

[49] Slamon DJ, Leyland-Jones B, Shak S, Fuchs H, Paton V, Bajamonde A (2001). Use of chemotherapy plus a monoclonal antibody against HER2 for metastatic breast cancer that overexpresses HER2. N Engl J Med.

