# Supplementary material for: Transmembrane TNF-alpha promotes chemoresistance in breast cancer cells
Source: Oncogene. 2018 Mar 21;37(25):3456–70. doi: 10.1038/s41388-018-0221-4 (PMC6013421; doi:10.1038/s41388-018-0221-4)
Supplement: Supplementary file 1 — Supplementary Data(DOC 2116 kb) [file 41388_2018_221_MOESM1_ESM.doc]

**Transmembrane TNF-alpha promotes chemoresistance in breast cancer cells**

Zunyue Zhang^1§^, Guohong Lin^1§^, Yujing Yan^2^, Xiang Li^2^, Yibing Hu^2^, Jing Wang^1^, Bingjiao Yin^1^, Yaqun Wu^2^, Zhuoya Li^1*^ and Xiang-Ping Yang^1*^

^1^Department of Immunology, School of Basic Medicine, Tongji Medical College, Huazhong University of Science and Technology, Wuhan 430030, China

^2^Department of Thyroid and Breast Surgery, Tongji Medical College, Tongji Hospital, Huazhong University of Science and Technology, Wuhan 430030, China

**Supplementary Materials and Methods**

**Immunohistochemistry staining.** 4-μm thick, formalin fixed and paraffin-embedded tumor tissue sections from the patients or animals were de-paraffinized in xylene and rehydrated in graded ethanol. Following heat-mediated antigen retrieval, the sections were incubated with primary antibodies including tmTNF-α, phosphorylated NF-κB p65, GST-π, or BCRP, followed by a 1-h incubation with biotin-conjugated antimurine IgG antibody (Boyao Biotechnology, Wuhan, China) and a 20-min incubation with peroxidase-labeled streptavidin. As a negative control, isotype antibody was substituted for the primary antibody. Color was developed for a 5-min reaction to the substrate diaminobenzidine. Tongji Qianping Image Analysis Software was used to determine the positive cell ratio (integral optical density value/integral area).

**Cell proliferation and clonogenic assays.** To test cell proliferation, CCK8 kit (Dojindo Laboratories, Kumamoto, Japan) was used according to manufacturer’s protocol. After incubation for 0 h, 24 h, 48 h and 72 h, CCK8 was added to cell culture and incubated for another 2 h. OD was measured at 450 nm on a microplate reader (Tecan, Grodig, Austria).

For the clonogenic assay, 500 cells/well were seeded in 6-well plates and grown in RPMI 1640 containing 10 % FBS. After 14 days, cells were fixed with 1% paraformaldehyde and stained with 0.5 % crystal violet (Sigma, USA). Colonies (diameter > 40 μm) in the dishes were counted. Clonogenic survival was calculated by the following formula: Clonogenic survival (%) = (Clone number of experimental group /Clone number of control group) X 100%

**Cell migration and invasion assays.** Scratch wound assay was used to test cell migration. After the formation of cell monolayer, the cells were scratched with a 200 μl sterile pipette tip, and cultured again for up to 24 h with 0.1% FBS RPMI 1640 medium after washing. Images were captured at 0 h and 24 h for the same scratched region under a phase-contrast microscope. Areas in the scratch were quantitatively analysed by using Image Pro-plus (IPP) software (Media Cybernetics, Rockville, MD, USA).

％wound closure = (wound width at 0 h – wound width at 24 h) X 100

wound width at 0 h

Transwell invasion assay was performed using Pore-occluding BD Matrgel Matrix-coated BD FluoroBlok Insert System (BD biosciences). Briefly, 5 X 10^4^cells in serum-free RPMI 1640 medium were placed into the upper chamber of matrigel matrix- coated insert. Media containing 15 % FBS were added to the lower chamber. After a 24-h incubation, the cells remaining on the upper side of membrane were removed with a cotton swab, while the invasive cells that had degraded matrix and crossed the membrane pores to its underside were fixed with 1% paraformaldehyde for 30 min, stained with 0.5 % crystal violet for 15 min (Sigma, USA), imaged and counted for 5 views under an inverted microscope (Canon, Japan).

**Flow cytometry assay.** Breast cancer cell lines were stained with antibodies specific to tmTNF-α, MRP-1, BCRP, MGMT, P-gp, GST-π or TOP2a or isotype control for 45 min at 4°C as described previously.^1^ Fixation/Permeabilization Concentrate and Diluent (eBioscience, USA) was used for intracellular or nuclear antigen staining. After washing, the cells were incubated with PE-conjugated secondary antibodies (Jackson biotech, USA) for 45 min at 4°C.

Cell cycle analysis was performed by quantification of DNA content in sub-G1 population. Briefly, the cells were fixed with 70% ethanol and stained with a solution containing 20 μg/ml propidium iodide (Sigma, USA) and 50 μg/ml RNase for 30 min (Sigma, USA). The stained cells were analyzed with an LSR II flow cytometer (Becton Dickinson, San Jose, CA) using BD FACS Diva software.

**DNA fragmentation analysis.** Breast cancer cells were lysed after a 24 h treatment with DOX. DNA fragmentation was determined in supernatant of lysate using a cellular DNA fragmentation ELISAPLUS kit (Roche Applied Science, USA) according to the manufacturer's instructions. OD value at 405 nm was measured on a microplate reader (Tecan, Austria).

**Reverse transcription PCR (RT-PCR) and quantitative real-time PCR (qPCR).** Total RNA was extracted from cells treated with or without DOX 3 μM for 24 h using Trizol (Invitrogen, USA), according to the manufacturer's instructions. 2 μg of the total RNA was reversely transcribed into first-strand cDNA using the Transcript First-Strand cDNA Synthesis SuperMix (TransGen, Beijing, China). Gene expression of human tmTNF-α and GAPDH was determined by RT-PCR with 2 X Es Taq MasterMix (Dye) (Cowin Biosciences, Beijing, China). The reactions were performed as follows: 3 min at 94°C, then followed by 30 s at 94°C, 30 s at 58°C and 40 s at 72°C for 35 cycles.

UltraSYBR-based (Cowin Biosciences, Beijing, China) qPCR was performed using primers for Bcl-X_L_, BAX, GST-π and β-actin (listed in Supplementary table S1). The PCR reactions were performed as follows: 5 min at 95°C, then followed by 15 s at 95°C, 20 s at 58°C and 20 s at 72°C for 40 cycles. Levels of mRNA were analyzed using the 2^-ΔΔCt^ method with Mx3000 software (Stratagene), and normalized with β-actin.

**Immunoblotting assay.** Cultured cells or fresh-frozen tumor tissues were lysed for 30 min with RIPA lysis buffer (50 mM Tris-HCl pH 7.4, 150 mM NaCl, 1% Nonidet P-40 and 0.5% sodium deoxycholate) containing protease inhibitors 0.5 mM PMSF, 5 mg/ml aprotinin, and 5 mg/ml leupeptin. Total protein was obtained after centrifugation at 15,000 rpm for 15 min at 4°C. Cytosolic and nuclear proteins were isolated with a commercial kit (Boster, Wuhan, China). Protein was separated by SDS-PAGE electrophoresis and transferred onto a PVDF membrane using a semi-dry transfer system (BioRad Laboratories, Hercules, CA, USA). Then the membranes were probed with primary antibodies against total ERK1/2, phosphorylated ERK1/2, IκB-α, p65, cIAP1, cIAP2, XIAP, BAX, Bcl-X_L_, GST-π, LamB 1, β-actin and tmTNF-α (listed in Supplementary table S2) as described previously^1^ after blockage overnight with 5% non-fat dry milk in PBS-Tween 20 (0.05%). An HRP-conjugated secondary antibody against rabbit or mouse IgG was used to detect the antigen-antibody complexes (Feiyi Biotech, Wuhan, China). The enhanced chemiluminescence (ECL) kit (Pierce, Rockford, USA) was used to visualize the antibody reaction.

**ELISA-based NF-κB activity assay.** NF-κB activity was detected with ELISA as previously described.^2^ Briefly, cells were lysed in lysis buffer containing a protease inhibitor cocktail (Calbiochem, San Diego, CA, USA). Two single-stranded oligonucleotide chains, 5′-AGTTGAGGGGACTTTCCCAGGC-C-(C)34-C-3-bio′, which is biotinylated at the 3′ end, and 5′-GCCTGGGAAAGTCCCCTCAACT-3′ were synthesized (Sangon, Shanghai). The double-stranded probe was formed by mixing two chains at a ratio of 1:1, denaturing at 94°C for 10 min, and then annealing at RT. Then the probe was added to streptavidin-coated 96-well at a final concentration of 2 pM. 5 μg of total protein mixed with binding buffer was added and incubated for 1 h at room temperature. The NF-κB activity was detected using a mAb against NF-κB p65 (Santa Cruz Biotechnology, Santa Cruz, CA, USA), followed by a peroxidase-conjugated secondary antibody (Santa Cruz, CA) and the substrate tetramethylbenzidine (Shanghai Lizhu Dongfeng Biotechnology, Shanghai). Quantification was performed via colorimetric readout of absorbance at 450 nm on a microplate reader (Tecan, Austria).

**Intracellular DOX** **measurement.** Cells were treated with DOX for 24 h. Thereafter, the cells were washed three times with PBS. The fluorescence intensity of intracellular DOX was analyzed on LSR II flow cytometer (Becton Dickinson, San Jose, CA) using BD FACS Diva software.

**Reference**

1 Yu M, Zhou X, Niu L, Lin G, Huang J, Zhou W *et al* (2013). Targeting transmembrane TNF-alpha suppresses breast cancer growth. *Cancer research* **73:** 4061-4074.

2 Jin S, Lu D, Ye S, Ye H, Zhu L, Feng Z *et al* (2005). A simplified probe preparation for ELISA-based NF-kappaB activity assay. *Journal of biochemical and biophysical methods* **65:** 20-29.

**Supplementary Figures and Legends**


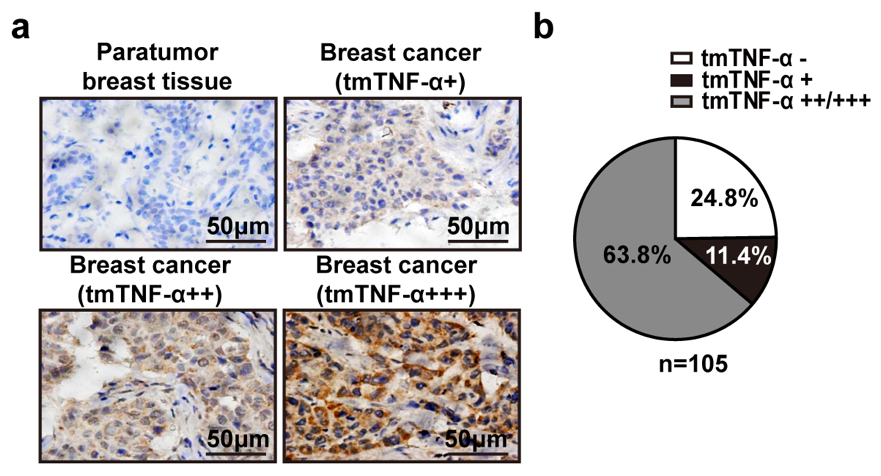


**Supplementary Figure 1. tmTNF-α overexpressed in breast cancer tissues.** (**a**) Immunohistochemically examination of tmTNF-α expression (from + to +++) on paratumoral (n=20) or tumoral tissue sections (n=105) from patients with ductal breast cancer (magnification, X40). Images are representative and the brown staining represents tmTNF-α expression. (**b**) Negative, weak (+) or strong (++/+++) positive rate of tmTNF-α in 105 patients with breast cancer.


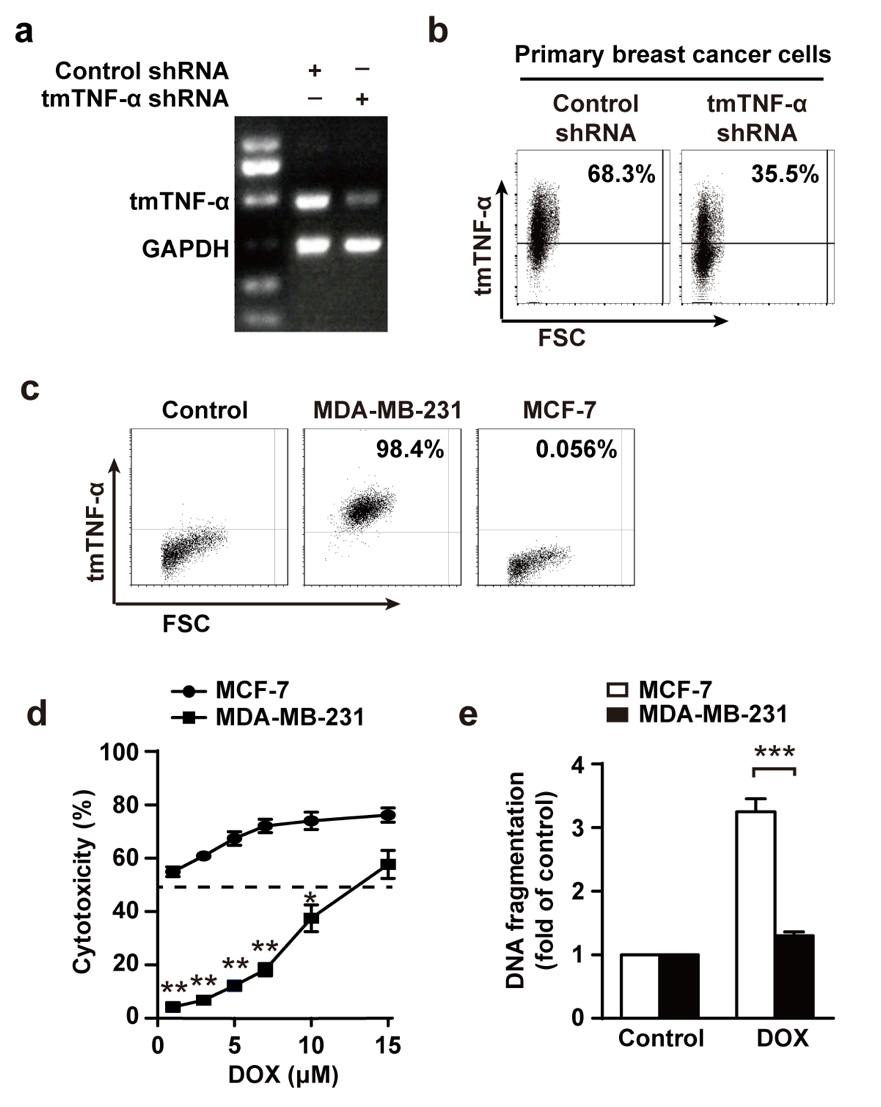


**Supplementary Figure 2. tmTNF-α is associated with DOX resistance of breast cancer.** (**a-b**) Primary human breast cancer cells were isolated from patients with high expression of tmTNF-α and transfected with control or tmTNF-α shRNA for 48 h. (**a**) TNF-α mRNA transcription shown by agarose gel electrophoresis, following RT-PCR. (**b**) FACS analysis of tmTNF-α expression on the cell surface. (**c**) FACS analysis of tmTNF-α expression in MDA-MB-231 and MCF-7 cells. (**d**) A 24-h cytotoxicity of DOX in indicated concentrations to MDA-MB-231 and MCF-7 cells. (**e**) DNA fragmentation of MCF-7 and MDA-MB-231 cells treated with 3μM DOX for 24 h detected by ELISA. Data are represented as mean ± SEM of at least three independent experiments. **p* ˂ 0.05, ***p* ˂ 0.01, ****p* ˂ 0.001


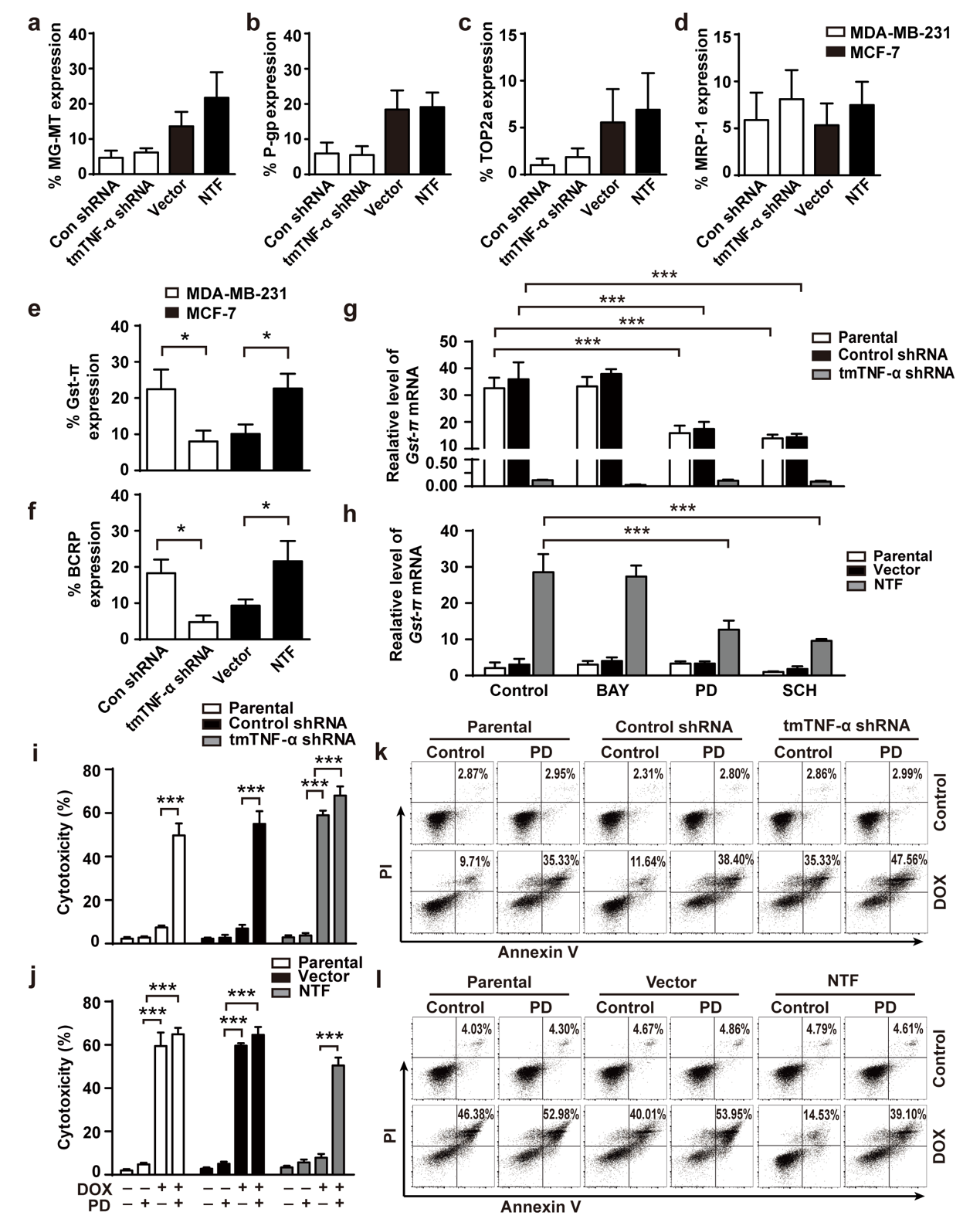


**Supplementary Figure 3. Drug-resistant molecules are involved in tmTNF-α-mediated doxorubicin resistance.** FACS analysis of expression of MGMT (**a**), P-gp (**b**), TOP2a (**c**), MRP-1 (**d**), GST-π (**e**), BCRP (**f**) in MDA-MB-231 transfected with control or tmTNF-α shRNA or MCF-7 cells transfected with empty vector or NTF. (**g** and **h**) Real-time PCR analysis of GST-π in parental or shRNA-transfected MDA-MB-231 cells and in parental or NTF-transfected MCF-7 cells treated with 10 μM BAY117082（BAY）or 10 μM PD98059（PD）for 24 h. (**i-l**) MDA-MB-231 cells or shRNA-transfected MDA-MB-231 cells and MCF-7 cells or NTF-transfected MCF-7 cells were pretreatment of 10 μM PD98059 for 30 min and then incubated with DOX (3 μM) for another 24 h. Cytotoxicity or apoptosis (representative cytogram) was determined by MTT assay (**i** and **j**) or Annexin V/PI staining (**k** and **l**). Data are represented as mean ± SEM of three independent experiments. **p* ˂ 0.05, ****p* < 0.001


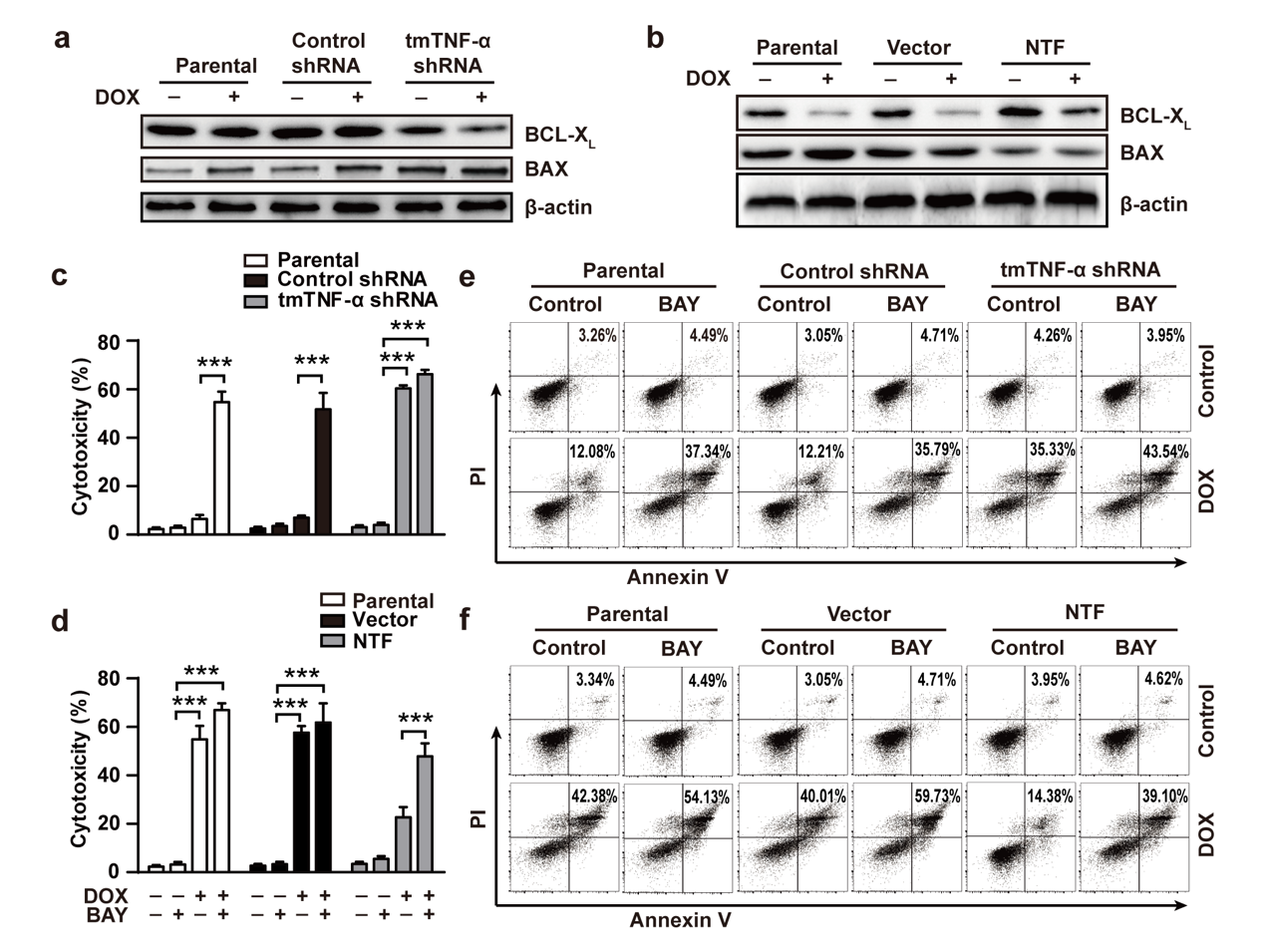


**Supplementary Figure 4. NF-κB is required for tmTNF-α-mediated DOX resistance.** Parental or shRNA-transfected MDA-MB-231 cells or parental NTF-transfected MCF-7 cells were treated with or without 3 μM DOX for 24 h. **(a and b)** Immunoblotting analysis of levels of Bcl-X_L_ and BAX. β-actin served as a loading control. **(c-f)** Cytotoxicity and apoptosis (representative cytogram) were determined in parental or shRNA-transfected MDA-MB-231 cells (**c** and **e**) or in parental or NTF-transfected MCF-7 cells (**d** and **f**) treated with or without 10 μM BAY117082（BAY）for 30 min prior to addition of DOX. Data are represented as mean ± SEM of three independent experiments. ****p* < 0.001

**Supplementary Tables**

**Table S1: Sequences of primers for RT-PCR or Real-Time PCR**

| **Gene** | **Primers** | **Length** |
| --- | --- | --- |
| Bcl-X_L_ | F:5'GTAGTGAATGAACTCTTTCGGGATGG3' | 288bp |
|  | R:5'AGCCACAGTCATGCCCGTCAGG3' |  |
| BAX | F:5'CCCGAGAGGTCTTTTTCCGAG3’ | 155bp |
|  | R:5'CCAGCCCATGATGGTTCTGAT3’ |  |
| GST-π | F:5'GAGGAGGTGGTGACCGTGGAGA3’ | 127bp |
|  | R:5'GGTGACGCAGGATGGTATTGGA3’ |  |
| β-actin | F:5'CCCATCTACGAGGGGTATGC3’ | 149bp |
|  | R:5'TTAATGTCACGCACGATTTC3' |  |
| TNF-α | F:5'TCTGCCTGCTGCACTTTGGA3' | 707bp |
|  | R:5'CTGGTAGGAGACGGCGATGC3' |  |
| GAPDH | F:5'ACCACAGTCCATGCCATCAC3' | 500bp |
|  | R:5'TCCACCACCCTGTTGCTGTA3' |  |

F: Forward; R: Reverse

**Table S2: Antibody information**

| **Antibody** | **Suppliers** | **Catalog Number** |
| --- | --- | --- |
| tmTNF-α | House made |  |
| MRP-1 | abcam | ab24102 |
| MGMT | abcam | ab39253 |
| P-gp | abcam | ab8189 |
| BCRP | Millipore | MAB4146 |
| TOP2a | Cell signaling | 4744 |
| GST-π | abcam | ab135535 |
| IκB-α | Santa Cruz | sc-371 |
| p65 | Santa Cruz | sc-8008 |
| cIAP1 | Santa Cruz | sc-7943 |
| cIAP2 | Santa Cruz | sc-7944 |
| Bcl-X_L_ | Santa Cruz | sc-7195 |
| XIAP | Boter | BA2620 |
| BAX | Boter | BA0315-2 |
| LamB 1 | Proteintech | 23498-1-AP |
| total ERK1/2 | SAB | #29162 |
| phosphorylated ERK1/2 | Cell signaling | #4370 |
| β-actin | Abbk | #A01010 |
| GAPDH | Abbk | #A01020 |
